# DEMETER: efficient simultaneous curation of genome-scale reconstructions guided by experimental data and refined gene annotations

**DOI:** 10.1093/bioinformatics/btab622

**Published:** 2021-09-02

**Authors:** Almut Heinken, Stefanía Magnúsdóttir, Ronan M T Fleming, Ines Thiele

**Affiliations:** School of Medicine, National University of Galway, H91 TK33 Galway, Ireland; Ryan Institute, National University of Galway, H91 TK33 Galway, Ireland; Center for Molecular Medicine, University Medical Center Utrecht, 3584 CX Utrecht, The Netherlands; School of Medicine, National University of Galway, H91 TK33 Galway, Ireland; Leiden Academic Centre for Drug Research, Leiden University, 2333 CC Leiden, The Netherlands; School of Medicine, National University of Galway, H91 TK33 Galway, Ireland; Ryan Institute, National University of Galway, H91 TK33 Galway, Ireland; Division of Microbiology, National University of Galway, H91 TK33 Galway, Ireland; APC Microbiome Ireland, University College Cork, T12 K8AF Cork, Ireland

## Abstract

**Motivation:**

Manual curation of genome-scale reconstructions is laborious, yet existing automated curation tools do not typically take species-specific experimental and curated genomic data into account.

**Results:**

We developed Data-drivEn METabolic nEtwork Refinement (DEMETER), a Constraint-Based Reconstruction and Analysis (COBRA) Toolbox extension, which enables the efficient, simultaneous refinement of thousands of draft genome-scale reconstructions, while ensuring adherence to the quality standards in the field, agreement with available experimental data and refinement of pathways based on manually refined genome annotations.

**Availability and implementation:**

DEMETER and tutorials are freely available at https://github.com/opencobra.

**Supplementary information:**

Supplementary data are available at *Bioinformatics* online.

## 1. Introduction

The Constraint-Based Reconstruction and Analysis (COBRA) approach relies on genome-scale metabolic reconstructions that have been curated based on genomic, biochemical and physiological data, a laborious process consisting of 96 steps (Thiele and Palsson, 2010). On the other hand, existing automated reconstruction pipelines, such as ModelSEED (Henry *et al.*, 2010), provide limited support for curation based on organism-specific experimental and genomic data.

Here, we present Data-drivEn METabolic nEtwork Refinement (DEMETER), a reconstruction pipeline that enables the efficient and simultaneous refinement of thousands of draft genome-scale reconstructions. DEMETER specializes in reconstructing human-associated microbes, which previously enabled the reconstruction of 773 gut microbes, AGORA (Magnusdottir *et al.*, 2017), as well its expansion, AGORA2, accounting for 7206 human microbial strains (Heinken *et al.*, 2020). The refinement of draft reconstructions in DEMETER is guided by a wealth of experimental data, such as carbon sources, fermentation pathways, and growth requirements, for over 1000 species, as well as by strain-specific comparative genomic analyses. Hence, DEMETER ensures the resulting refined reconstructions capture known traits of the target organisms.

## 2. Features

**Fig. 1.**
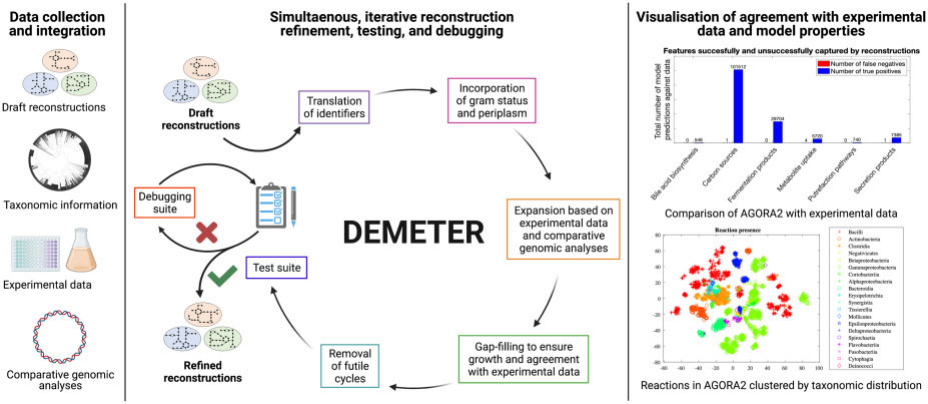
Overview of the DEMETER workflow consisting of (i) data collection and integration, (ii) simultaneous refinement, testing and debugging of the draft reconstructions, and (iii) visualization of test results and computation of model properties. Created with BioRender.com

The DEMETER pipeline consists of three main steps: (i) data collection and integration, (ii) draft reconstruction refinement, testing and debugging, and (iii) computation of model properties (Fig. 1).

### 2.1. Data collection and integration

The minimal prerequisite is the availability of a sequenced genome for the organisms of interest. An essential step is the generation of draft genome-scale reconstructions, e.g., using ModelSEED (Henry *et al.*, 2010) or KBase (Arkin *et al.*, 2018). Where possible, gram status and species-specific experimental data are propagated to the target organisms. Moreover, strain-specific comparative genomic analyses retrieved from PubSEED subsystems (Aziz *et al.*, 2012) can be mapped to DEMETER.

### 2.2. Refinement step

During the refinement step, the draft reconstructions are systematically improved (Fig. 1). Briefly, the following steps are performed:

- Translation from ModelSEED to Virtual Metabolic Human (Noronha *et al.*, 2019) reaction and metabolite nomenclature.
- Curation of the biomass objective function based on gram status and, where appropriate, generation of a periplasmatic compartment.
- Inclusion of species-specific pathways for carbon source utilization, fermentation products, and consumed and secreted metabolites.
- Refinement of pathways and gene-protein-reaction associations based on strain-specific comparative genomic analyses.
- Removal of futile cycles to ensure thermodynamic feasibility.
- Gap-filling to ensure growth and agreement with provided experimental data, including complex and defined media.
- Quality-controlled rebuilding of the resulting refined reconstruction.

### 2.3. Test and debugging suite

To ensure high quality and predictive potential of the refined reconstructions generated by DEMETER, a test suite is provided that performs systematic quality control and quality assurance (Fig. 1). Any errors are subsequently corrected through a provided automated debugging suite. Some reconstructions may require additional manual inspection.

### 2.4. Analysis of model properties

To elucidate how metabolic traits are spread across strains, model features including reaction and metabolite content, metabolite uptake and secretion potential, and internal metabolite biosynthesis potential are computed and subsequently visualized. Taxonomically close strains reconstructed by DEMETER are also similar in their reaction content (Fig. 1).

## 3. Implementation and code availability

DEMETER is written in MATLAB (Mathworks, Inc.) and is freely available at the COBRA Toolbox GitHub https://github.com/opencobra/cobratoolbox (Heirendt *et al.*, 2019). A comprehensive tutorial in form of a MATLAB live script (Supplementary File S1) is provided at https://github.com/opencobra/COBRA.tutorials.

## 4. Discussion

Refined reconstructions built through DEMETER adhere to the quality standards in the COBRA field and capture the known metabolic features of the target organisms. Hence, they are suitable for predictive modeling studies, such as the construction and interrogation of personalized microbiome models. Note that while DEMETER was initially developed for the human microbiome, it can be applied to any bacterial or archaeal species.

## Funding

This study was funded by grants from the European Research Council (ERC) under the European Union’s Horizon 2020 research and innovation programme [757922 to I.T.] and by the National Institute on Aging grants [1RF1AG058942-01 and 1U19AG063744-01].

*Conflict of Interest*: none declared.

## Supplementary Material

btab622_Supplementary_Data
