# Supplementary material for: DEMETER: efficient simultaneous curation of genome-scale reconstructions guided by experimental data and refined gene annotations
Source: Bioinformatics. 2021 Sep 2;37(21):3974–5. doi: 10.1093/bioinformatics/btab622 (PMC8570805; doi:10.1093/bioinformatics/btab622)
Supplement: btab622_Supplementary_Data [file btab622_supplementary_data.pdf]

Refinement of genome-scale reconstructions through the DEMETER pipeline

Author: Almut Heinken, PhD, National University of Ireland Galway

01/2021

Introduction

This tutorial introduces the semi-automated reconstruction pipeline DEMETER (Data-drivEn METabolic nEtwork Refinement) [1] and its use for semi-automated, data-driven reconstruction of microbial strains. DEMETER was first applied to the reconstruction of 773 human gut microbial organisms [2]. More recently, DEMETER was used to reconstruct 7,206 human microbial strains, resulting in AGORA2, an update to AGORA both in size and scope [3]. While DEMETER was built with reconstructing human gut microbial reconstructions in mind and is tailored towards this purpose, it can be applied to any types of bacterial or archaeal microorganisms. The minimal prerequisite is the availability of a sequenced genome for the target organism. The tutorial demonstrates how to use DEMETER to build a refined genome-scale reconstruction for one or more target organisms from a draft reconstruction retrieved from KBase. Steps include input data collection, input data integration, running the refinement pipeline, and testing the refined reconstruction against the input data. Steps Figure 1 summarizes the steps used to create a curated reconstruction.

DEMETER has been designed to handle large-scale tasks of refining hundreds or even thousands of draft reconstructions. This may be time-consuming. It is recommended to use parallel computing for improved efficiency. If the pipeline run is interrupted, it can be rerun and already performed steps will be skipped.

Requirements

This tutorial requires the Parallel Computing Toolbox, Bioinformatics Toolbox, and Statistics and Machine Learning Toolbox add-ons in MATLAB. DEMETER relies on files located in the COBRA/papers repository. Please ensure that the COBRA/papers folder is up to date and the COBRA Toolbox including the papers folder are in the MATLAB path.

Start the tutorial by initializing the COBRA Toolbox.

```
initCobraToolbox
```

Please ensure that the COBRA/papers folder is up to date and in the MATLAB path as DEMETER depends on files located in COBRA/papers/demeter.

Set a solver (recommended: IBM CPLEX)

```
solverOK=changeCobraSolver('ibm_cplex','LP');
```

```
> changeCobraSolver: IBM ILOG CPLEX interface added to MATLAB path.
> The solver compatibility is not tested with MATLAB R2019b.
```

```
global CBTDIR
```

Step 1: Data collection and data integration

This part explains how to collect input data and convert it into a format readible by the pipeline. The minimal input for building a refined reconstruction is a KBase draft reconstruction. Recommended inputs include taxonomic information on the target organism, experimental data if the species is not yet found in the already collected data, and comparative genomic analyses.

1.1. KBase draft reconstructions-required

Create KBase draft reconstruction(s) for the target organism(s) by using the apps at kbase.us/. A template narrative to facilitate this is available at https://narrative.kbase.us/narrative/ws.82195.obj.8. The narrative assumes that the genomes to reconstruct are available as FASTA files and imported into the Staging Area as a zipped file. Alternatively, genomes available in KBase can be directly imported into the Narrative. Note that it will not be possible to reconstruct organisms without a sequenced genome. Export the generated draft reconstructions by downloading them.

Next, please place all draft reconstructions that you want to refine into one folder. For the sake of this tutorial, we will use four AGORA2 strains as well as six example strains that were randomly chosen from genomes present at KBase and are not present in AGORA2. Draft reconstructions for these strains have already been retrieved from KBase and placed into the folder cobratoolbox/papers/2021_demeter/exampleDraftReconstructions. Alternatively, they are available in the template narrative https://narrative.kbase.us/narrative/81207.

To set the path to the example draft reconstructions, enter the code:

```
draftFolder = [CBTDIR filesep 'papers' filesep '2021_demeter' filesep
'exampleDraftReconstructions'];
```

1.2. Taxonomic and strain information

Information on the taxonomic classification and organism properties (e.g., gram status, oxygen status, metabolism type, pathogenicity) has been collected for all 7,206 AGORA2 strains and is available in the file "input/AGORA2_infoFile.xlsx". The AGORA2 reference organism data is continuously being updated to the best of our abilities. It is highly recommmended to collect taxonomic information for your organism as this enables the propagation of experimental data from related strains that are already present in AGORA2. Moreover, it allows for the curation of the biomass objective function for gram status of the organism and inclusion of a periplasm compartment where appropriate. Taxonomic information can be retrieved from GTdb: https://gtdb.ecogenomic.org/ or NCBI Taxonomy: https://www.ncbi.nlm.nih.gov/taxonomy/. To provide taxonomic information for your organisms, prepare a file in the same format as cobratoolbox/papers/2021_demeter/example_infoFile.xlsx. Please provide the path to the file with taxonomic information as the variable infoFilePath.

```
infoFilePath = [CBTDIR filesep 'papers' filesep '2021_demeter' filesep
'example_infoFile.xlsx'];
```

IMPORTANT: The IDs in the column 'MicrobeID' need to match the names of the resulting refined reconstructions exactly. Prepare the file with taxonomic information accordingly. During the pipeline run, the names of draft reconstruction files will be adapted. To find out what the names of the refined reconstructions will be, run the function

```
refinedModelIDs = printRefinedModelIDs(draftFolder);
```

1.3. Propagating existing experimental data

The DEMETER pipeline contains experimental data for >1,500 microbial species for (i) carbon sources, (ii) fermentation pathways, (iii) growth requirements, (iv) consumed metabolites, and (v) secreted metabolites. The data are stored in MATLAB-readable format in the input folder as the files CarbonSourcesTable.txt, FermentationTable.txt, NutrientRequirementsTable.txt, secretionProductTable.txt, and uptakeTable.txt. If a strain related to your organism is already present in the DEMETER resource and has data available, this data can be propagated to your new organism. This requires taxonomic information organized in a table as described in the previous step. Note that you can still adapt the experimental data to strain-specific findings and add new data afterwards as needed. The input file with taxonomic information will also be supplemented with gram staining information if possible, which serves to additionally inform and refine the reconstructions. If no related organisms are already present in DEMETER from which gram status information can be propagated, gram status can be retrieved from literature searches or by querying the Integrated Microbial Genomes Database (https://img.jgi.doe.gov/) [4].

1.4. Using compatative genomic data as input data for DEMETER

It is possible to inform the reconstruction with comparative genomics data retrieved from PubSEED. This will require either retrieving publicly available spreadsheets from PubSEED or the generation of such spreadsheets through PubSEED (https://pubseed.theseed.org). Under https://pubseed.theseed.org/seedviewer.cgi?page=SubsystemSelect, over 1,000 curated subsystems for thousands of bacteria are available that can serve as input for DEMETER. See "Subsystem Spreadsheet" tab on PubSeed or cobratoolbox/papers/2021_demeter/exampleSpreadsheets for an example. To map the strains in subsystems to strains to reconstruct, the column PubSeedID needs to contain the translation to the respective IDs in the PubSeed spreadsheets.

Moreover, appropriate reactions in VMH nomenclature need to be mapped to each functional role in the spreadsheet (see "Functional Role" tab on PubSeed). Functional roles need to be provided as columns and mapped to corresponding reactions through the file cobratoolbox/papers/2021_demeter/input/InReactions.txt.

If comparative genomics data is available for your organism(s), please place it in a folder and provide the path to the folder as the variable spreadsheetFolder.

```
spreadsheetFolder = [CBTDIR filesep 'papers' filesep '2021_demeter' filesep
'exampleSpreadsheets'];
```

Additionally, the PubSEED IDs for each strain to refine, as well as the annotation version, need to be added to the "PubSeedID" and "Annotation version ID" columns, respectively, in the taxonomic information file as shown in example_infoFile.xlsx.

Define the path to a folder where the propagated data that will serve as input for DEMETER should be stored (optional). Otherwise, a default path will be used.

```
inputDataFolder = [pwd filesep 'InputData'];
```

To propagate available gram staining information, comparative genomics data, and experimental data to your organism(s), use the code

```
[infoFilePath,inputDataFolder] =
prepareInputData(infoFilePath,'inputDataFolder', inputDataFolder,
'spreadsheetFolder',spreadsheetFolder);
```

1.5. Manual gathering of experimental data as input data for DEMETER

In the folder inputDataFolder, you will find experimental data for your organism(s) retrieved and propagated from the already available data that had been collected for DEMETER. It is highly recommended that you manually inspect the propagated experimental data for your organism(s) at this point to verify it as organisms may differ on the strain level. Moreover, it is recommended that you perform additional literature searches to inform the refinement of your organism(s). As you can see from inspection of the newly generated text files, for three of the four new strains used in this tutorial, experimental data could be propagated from already reconstructed strains of the same species. For the fourth strain, Acetitomaculum ruminis DSM 5522, no related strain in present in DEMETER. To find experimental data on this organism, we go to PubMed (https://pubmed.ncbi.nlm.nih.gov/) and enter "Acetitomaculum ruminis" as a search term. This yields a published reference describing the metabolism of this species: PubMed ID 2500921, https://link.springer.com/article/10.1007/BF00416597. The paper reports that A. ruminis generates acetate through the acetogen pathway and uses formate and glucose as carbon sources. By converting this data into a MATLAB-readable format, we can ensure the associated pathways are present in the refined reconstruction.

To incorporate this data into the files with experimental data, use the following code.

Read the file with carbon source information.

```
data=readtable([inputDataFolder filesep 'CarbonSourcesTable.txt'], 'Delimiter',
'tab', 'ReadVariableNames', false);
data=table2cell(data);
```

Add information that glucose is used as a carbon source.

```
findRow=find(strcmp(data(:,1),'Acetitomaculum_ruminis_DSM_5522'));
findCol=find(strcmp(data(1,:),'D-glucose'));
data{findRow,findCol}='1';
writetable(cell2table(data),[inputDataFolder filesep
'CarbonSourcesTable'],'FileType','text','WriteVariableNames',false,'Delimiter','
tab');
```

Read the file with information on consumed metabolites.

```
data=readtable([inputDataFolder filesep 'uptakeTable.txt'], 'Delimiter', 'tab',
'ReadVariableNames', false);
data=table2cell(data);
```

Add information that formate is consumed.

```
findRow=find(strcmp(data(:,1),'Acetitomaculum_ruminis_DSM_5522'));
findCol=strcmp(data(1,:),'Formate');
data{findRow,findCol}='1';
writetable(cell2table(data),[inputDataFolder filesep
'uptakeTable'],'FileType','text','WriteVariableNames',false,'Delimiter','tab');
```

Read the file with fermentation product information.

```
data=readtable([inputDataFolder filesep 'FermentationTable.txt'], 'Delimiter',
'tab', 'ReadVariableNames', false);
data=table2cell(data);
```

Add information that the acetogen pathway is used.

```
findRow=find(strcmp(data(:,1),'Acetitomaculum_ruminis_DSM_5522'));
findCol=find(strcmp(data(1,:),'Acetogen pathway'));
data{findRow,findCol}='1';
writetable(cell2table(data),[inputDataFolder filesep
'FermentationTable'],'FileType','text','WriteVariableNames',false,'Delimiter','t
ab');
```

Alternatively, you can inspect the files manually and edit as needed.

To check the experimental data available that will be used for refinement for each strain to reconstruct, run the function:

```
curationStatus = getCurationStatus(infoFilePath,inputDataFolder,false);
```

2 indicates that experimental data is available to curate the reconstruction. 1 indicates that published literature is available but contains no usable data. 0 indicates no available experimental data.

Step 2: Iterative refinement

2.1. Running the DEMETER pipeline

With the experimental and comparative genomic data in place, the pipeline can be run. At this step, the draft reconstructions retreived previously from KBase or ModelSEED serve as the input data and are systematically curated. This results in a refined reconstruction for each strain in the draft reconstruction folder as a mat file and optionally as a SBML file. Moreover, a summary of refinement

steps, such as addition and removal of reactions during gap-filling, will be provided. Steps that are automatically carried out during this step include # translation of KBase reaction and metabolite to Virtual Metabolic Human [5] (https://www.vmh.life) nomenclature, # expansion of the reconstruction by pathways supported by experimental data, # refinement of pathways and gene rules against comparative genomic data, and # quality control ensuring e.g., thermodynamic feasibility.

REQUIRED INPUTS

Thefirst required input variable is the path to the folder containing the draft reconstructions (draftFolder defined above). Another required input is the path to the file with organism taxonomy (infoFilePath defined above).

OPTIONAL INPUTS

Define the number of workers for parallel computing.

```
numWorkers = 4;
```

Define a name for the reconstruction resource (default name will be used otherwise)

```
reconVersion = 'TutorialExample';
```

To run the pipeline, enter the code

```
[reconVersion,refinedFolder,translatedDraftsFolder,summaryFolder] =
runPipeline(draftFolder, 'infoFilePath', infoFilePath, 'inputDataFolder',
inputDataFolder, 'numWorkers', numWorkers, 'reconVersion', reconVersion);
```

Inspection of the output of the pipeline

Let us have a look at the results of the pipeline run. The refined reconstruction in mat format are located in the folder "RefinedReconstructions", and in SBML format in "RefinedReconstructions_SBML". The folder "TranslatedDraftReconstructions" contains the draft reconstructions with exchange reactions translated (no other changes were made to this version). The folder "RefinementSummary" contains a detailed overview of the gap-filling and expansion performed for each refined reconstruction. If the files "untranslatedMets.txt" and "untranslatedRxns.txt", are present and contain any entries, it is recommended to translate these metabolites and/or reactions. Go to Part 2.3 for more details.

2.2. Testing the refined reconstructions

DEMETER includes a comprehensive test suite that was developed to ensure good quality and agreement with known metabolic traits of the reconstructed strains in AGORA2 [2].

Define the folder where test results should be saved (optional, default folder will be used otherwise).

```
testResultsFolder = [pwd filesep 'TestResults'];
```

Biomass production

Test and plot whether all reconstructions can produce reasonable amounts of biomass on the Western diet and on "rich" medium (consisting of every metabolite the model can consume) aerobically and anaerobically.

```
notGrowing =
plotBiomassTestResults(refinedFolder,reconVersion,'translatedDraftsFolder',translatedDraftsFolder,'testResultsFolder',testResultsFolder, 'numWorkers',
numWorkers);
```

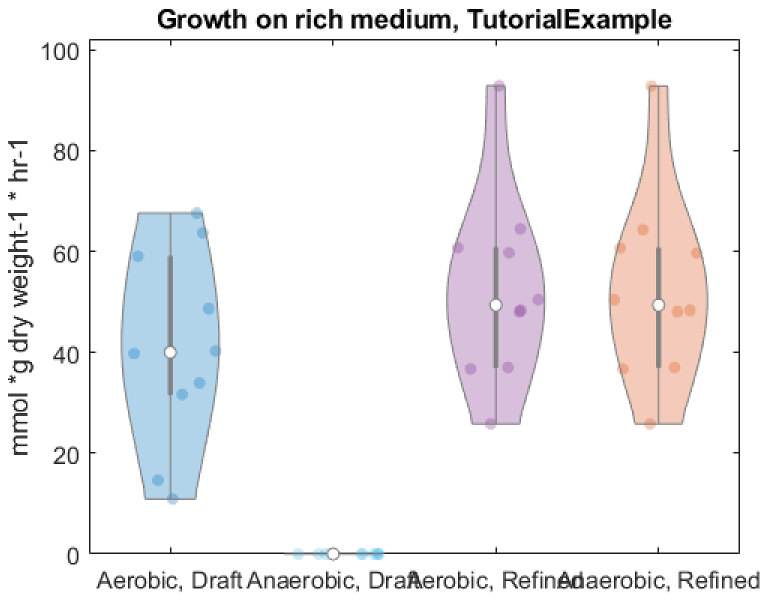

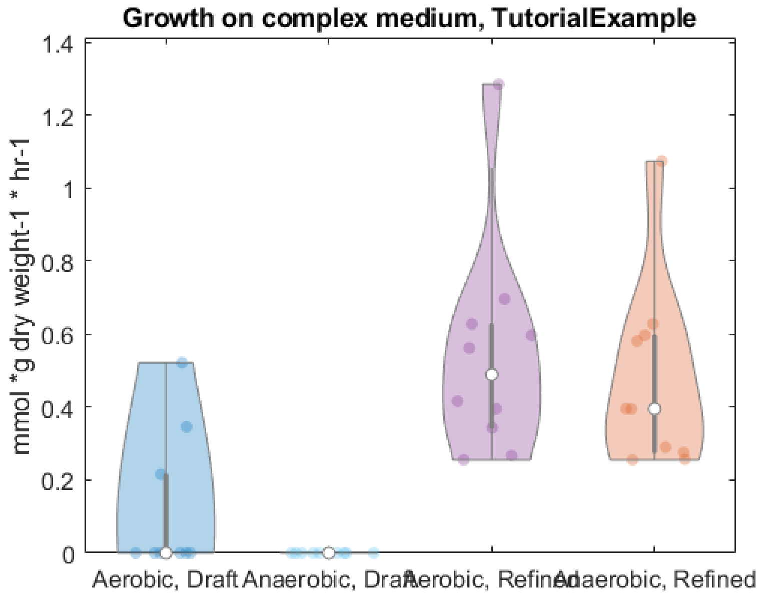


```
Report for draft models:
All models are able to produce biomass on rich medium.
10 models are unable to produce biomass on rich medium under anaerobic conditions.
7 models are unable to produce biomass on complex medium.
```

```
10 models are unable to produce biomass on complex medium under anaerobic conditions.
Report for refined models:
All models are able to produce biomass on rich medium.
All models are able to produce biomass on rich medium under anaerobic conditions.
All models are able to produce biomass on complex medium.
All models are able to produce biomass on complex medium under anaerobic conditions.
```

You can see that for the examples, all refined reconstructions produce biomass under anaerobic conditions and on Western diet while no draft reconstruction can grow anaerobically. If the refined reconstructions for your own organisms are unable to produce biomass under any conditions, proceed to Step 2.3 of the pipeline.

ATP production

% Test and plot whether all reconstructions produce reasonable amounts of ATP on the Western diet aerobically and anaerobically.

```
tooHighATP =
plotATPTestResults(refinedFolder,reconVersion,'translatedDraftsFolder',translatedDraftsFolder,'testResultsFolder',testResultsFolder, 'numWorkers', numWorkers);
```

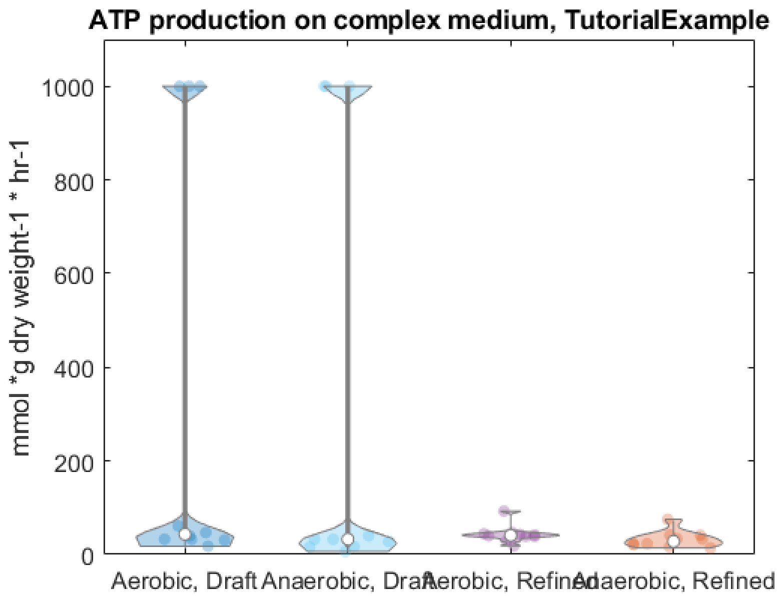


```
Report for draft models:
3  models produce too much ATP under aerobic conditions.
3  models produce too much ATP under anaerobic conditions.
Report for refined models:
All models produce reasonable amounts of ATP under aerobic conditions.
All models produce reasonable amounts of ATP under anaerobic conditions.
```

You can see that for the examples, all refined reconstructions produce realistic amounts of ATP on Western diet while the draft reconstructions produce near unlimited amounts of ATP. If the refined reconstructions for your own organisms produce too much ATP, proceed to Step 2.3 of the pipeline below.

QC/QA and test against available experimental and comparative genomic data

Run the test suite.

```
 [testResultsFolder,curationReport] = runTestSuiteTools(refinedFolder,
infoFilePath, inputDataFolder, reconVersion, 'translatedDraftsFolder',
translatedDraftsFolder, 'numWorkers', numWorkers, 'testResultsFolder',
testResultsFolder);
```

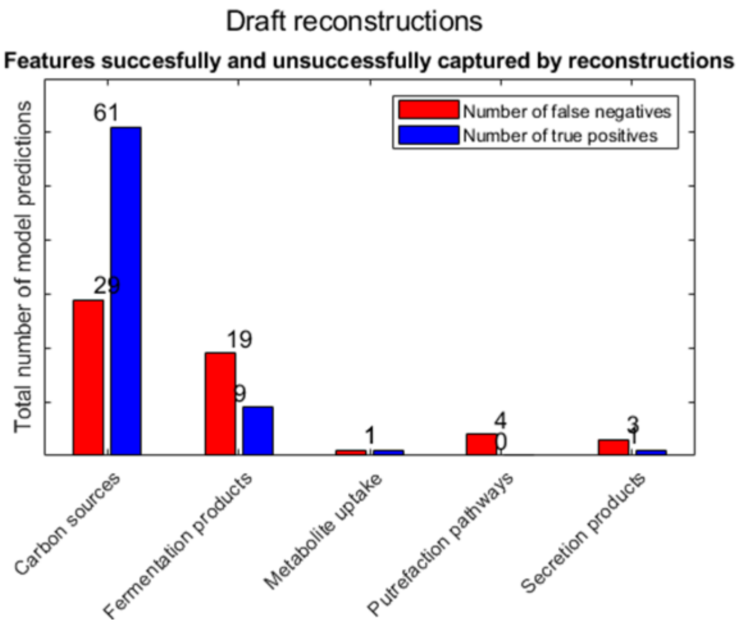

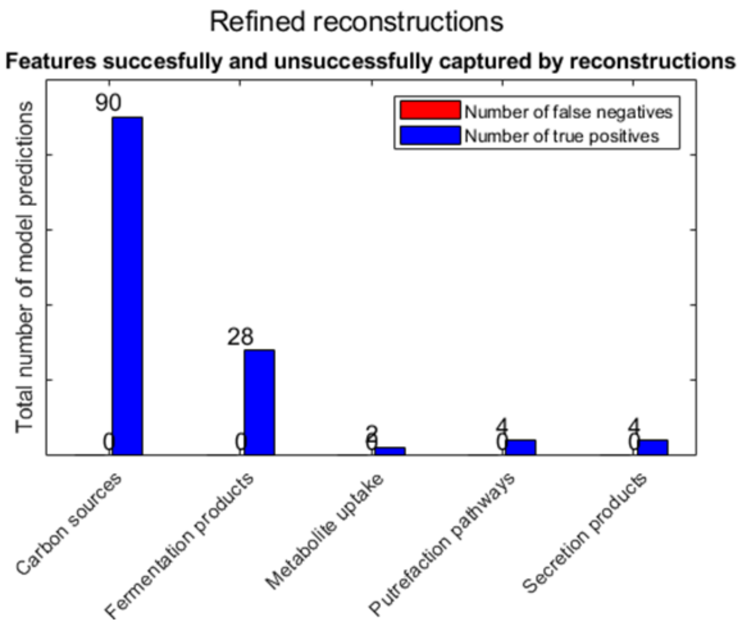

```
Finished refinement and testing of the project TutorialExample.
9 draft reconstructions have been refined.
Carbon source pathways were refined for 8 reconstructions.
100.00 % of reconstructions agree with all carbon source experimental data.
Fermentation pathways were refined for 9 reconstructions.
100.00 % of reconstructions agree with all fermentation experimental data.
Putrefaction pathways were refined for 1 reconstructions.
100.00 % of reconstructions agree with all putrefaction pathway data.
Secretion product pathways were refined for 2 reconstructions.
100.00 % of reconstructions agree with all secretion product experimental data.
Metabolite uptake pathways were refined for 2 reconstructions.
100.00 % of reconstructions agree with all metabolite uptake experimental data.
```

The variable curationReport contains a summary of the results of performed quality control and quality assurance tests.

The folder "TestResults" contains information on tests against the available experimental data that passed or failed the test suite. Carefully inspect these results. If there are any false negative predictions for your organism(s), proceed to Step 2.3 of the pipeline below.

To view the results for the tutorial example, run the following code.

Sensitivity of reconstructions for capturing the experimental data

```
testResults1 = readtable([testResultsFolder filesep reconVersion '_refined' filesep 'TutorialExample_Properties.xls'], 'ReadVariableNames', false);
testResults1 = table2cell(testResults1);
```

Fraction of refined reconstructions that agree with all experimental data

```
testResults2 = readtable([testResultsFolder filesep reconVersion '_refined' filesep 'TutorialExample_PercentagesAgreement.xls'], 'ReadVariableNames', false);
testResults2 = table2cell(testResults2);
```

The test suite also reports any cases of leaking metabolites, mass- and charge-imbalanced reactions, incorrectly formulated gene rules. Inspect these cases. Note that some mass- and charge-imbalanced reactions currently cannot be corrected.

2.3. Debugging the refined reconstructions

2.3.1. Translation from KBase to VMH nomenclature

To date, over 4,000 metabolites and over 6,700 reactions have been translated from KBase to VMH nomenclature. This includes most reactions commonly occuring in draft reconstructions for host-associated microbes. Especially for non-host-associated microbes, you may need to translate yet unmapped reactions and metabolites from KBase to VMH nomenclature. To find out if this is necessary, check the files "untranslatedMets.txt" and "untranslatedRxns.txt", if present, for any KBase metabolites and reactions that are not yet translated to VMH nomenclature. This requires the reconstruction tool rBioNet [6]. To prepare the use of rBioNet, run the function

```
createRBioNetDBFromVMHDB
```

Translating metabolites

If the files 'untranslatedMets.txt' contains metabolites, run the code

```
if isfile([summaryFolder filesep 'untranslatedMets.txt'])
[translatedMets]=translateKBaseToVMHMets('untranslatedMets.txt');
end
```

Carefully inspect the output 'translatedMets.txt'. If you are satisfied with the translation, add the KBase IDs with the corresponding VMH IDs to the file cobratoolbox/papers/2021_demeter/input/MetaboliteTranslationTable.txt.

Metabolites that could not be translated this way will need to be inspected manually. Try finding them in the VMH database by consulting KEGG (https://www.genome.jp/kegg) [7] or HMDB (https://hmdb.ca) [8] IDs. For metabolites that are not yet present in the VMH database, formulate a metabolite ID following the standards of the field as described in Thiele and Palsson [9]. Afterwards, add the new metabolites to the file cobratoolbox/papers/2021_demeter/input/MetaboliteDatabase.txt while ensuring that the format of the file is kept. Also add the KBase to VMH metabolite translation to the file cobratoolbox/papers/2021_demeter/input/MetaboliteTranslationTable.txt.

Translating reactions

If the files 'untranslatedRxns.txt' contains reactions, run the code

```
if isfile([summaryFolder filesep 'untranslatedRxns.txt'])
[translatedRxns]=propagateKBaseMetTranslationToRxns('untranslatedRxns.txt');
end
```

When translated from KBase to VMH nomenclature, some reactions will afterwards be mass-and charge-imbalanced. Especially the proton in the reaction will need to be adjusted.To test if any translated reactions are now unbalanced, use the reconstruction tool rBioNet [5].

Afterwards, run the command

ReconstructionTool

This opens an interface. Choose File -> Add Text File -> With Reactions -> Load text file.

Choose the text file translatedRxns.txt that was just generated. When it says "Metabolites not in Database", choose Yes. When it says "Unbalanced reactions", carefully inspect every reaction with unbalance charge or atoms. Adjust the formulas in the file translatedRxns.txt accordingly and save the file afterwards. Note that some mass- and charge-imbalanced reactions currently cannot be corrected.

Now we will check if the mass-and charge-balanced but untranslated reactions are already present in the VMH database but not yet translated. To check which of the translated reactions are already present in the VMH reaction database, run the function

```
 if isfile('translatedRxns.txt')
 [sameReactions,similarReactions] =
mapKBaseToVMHReactions('translatedRxns.txt');
 end
```

After verifying the content of the output variable sameReactions, add the KBase reaction IDs with the corresponding VMH reaction IDs to the file cobratoolbox/papers/2021_demeter/input/ReactionTranslationTable.txt

Note that multiple KBase reactions may translate to the same VMH reaction. Now inspect the content of the output variable similarReactions. Typically, this will contain reactions that are reversible in KBase but irreversible in VMH. It is optional but recommended that you also add these reactions to ReactionTranslationTable.txt.

For reactions that are not yet present in the VMH database, formulate a reaction ID following the standards of the field as described in Thiele and Palsson [8]. Import the reactions in ReconstructionTool as described above to ensure they are mass-and charge-balanced. Afterwards, add the new reactions to the file cobratoolbox/papers/2021_demeter/input/ReactionDatabase.txt

while ensuring that the format of the file is kept. Also assign subsystems to the reactions following the existing nomenclature in the database. Also add the KBase to VMH reaction translation to cobratoolbox/papers/2021_demeter/input/ReactionTranslationTable.txt.

We kindly request you also propagate this information back by submitting a pull request to COBRA/papers.

2.3.2 Debugging the reconstructions

After performing the data-driven refinement, there may still be reconstructions that cannot grow anaerobically and/or on Western diet, produce unrealistic amounts of ATP on Western diet, or do not agree with experimental data. Since DEMETER was built on the principle of inspecting patterns of reactions across many reconstructions, there may still be reconstructions (especially of non-gut organisms) with previously unobserved patterns that are not yet caught by the gap-filling functions in DEMETER.

Too inspect and attempt to correct these reconstructions, a suite of debugging tools is available. The suite will gap-fill the reconstructions that fail tests and re-test them afterwards.

To run the debugging suite, enter the code:

```
 [debuggingFolder,debuggingReport, fixedModels,
failedModels]=runDebuggingTools(refinedFolder,testResultsFolder,inputDataFolder,
reconVersion,'numWorkers',numWorkers);
```

All models passed all tests. Exiting debugging tools.

If any models still fail a test as indicated by the output variable failedModels, inspect the results in debuggingFolder -> Retest.

For models that still produce too much ATP, run the debugging function

futileCycleReactions = identifyFutileCycles(model);

Inspect the models that do not agree with experimental data manually to find out which steps of the biosynthesis pathway are blocked.

If this should be unsuccessful, feel free to open an issue at the COBRA Toolbox GitHub.

3.1 Analysis and visualization of model properties and features

Once refined reconstructions have been successfully created, the DEMETER pipeline also provides functions to retrieve properties of the reconstructions, e.g., average size and gene count and stochiometric consistency. Moreover, the pipeline enables the computation of reaction content, uptake and secretion profiles, and internal metabolite biosynthesis profiles per reconstruction and their subssequent clustering by taxon.

To run the computation and visualization of model properties, enter the code

```
 propertiesFolder = computeModelProperties(refinedFolder, infoFilePath,
reconVersion, 'numWorkers', numWorkers);
```

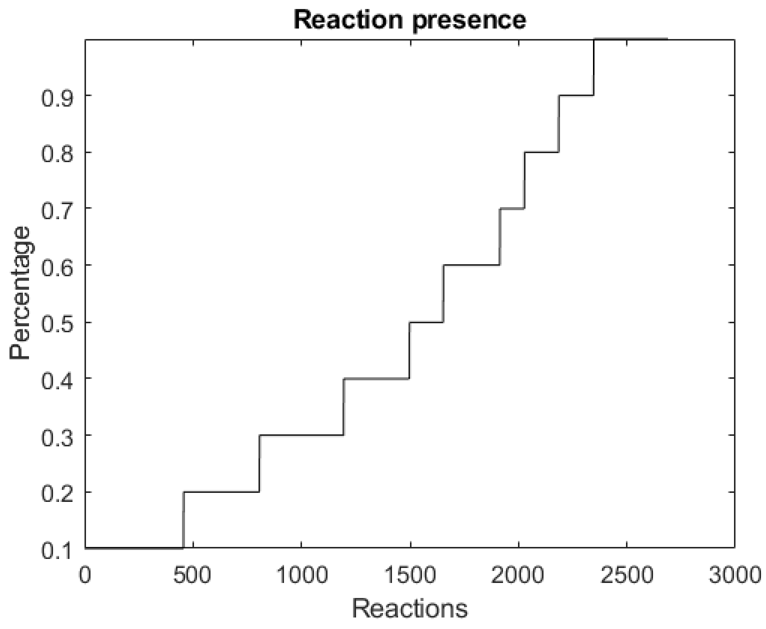

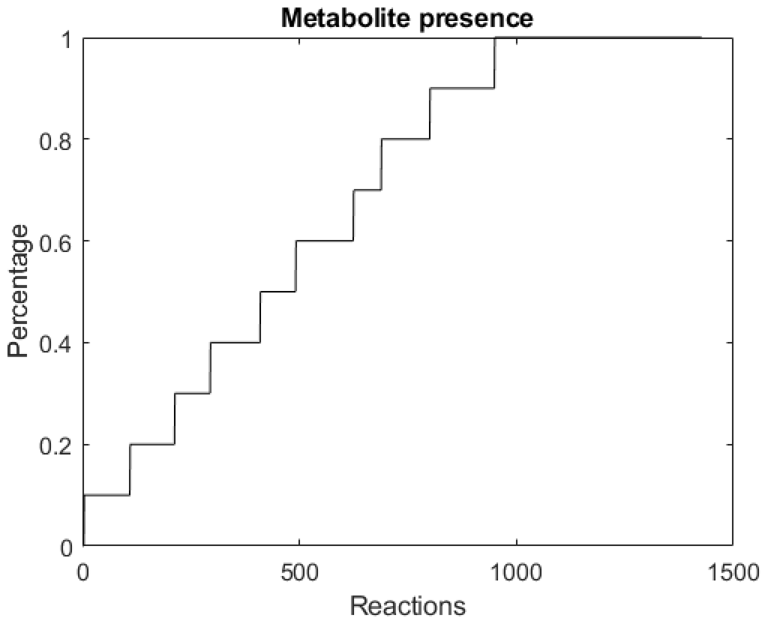

Metabolite presence
Percentage
Reactions
0
0.2
0.4
0.6
0.8
1
0
500
1000
1500

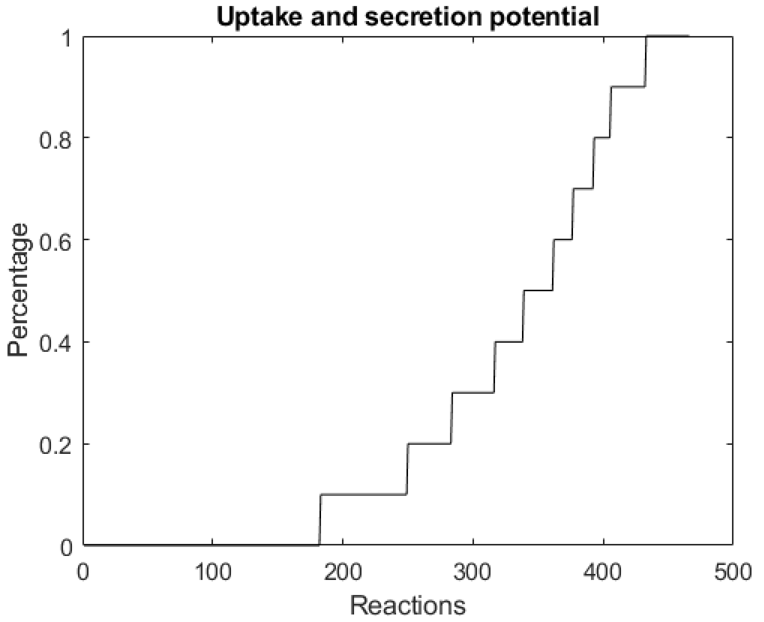

Uptake and secretion potential
Percentage
Reactions
0
0.2
0.4
0.6
0.8
1
0
100
200
300
400
500

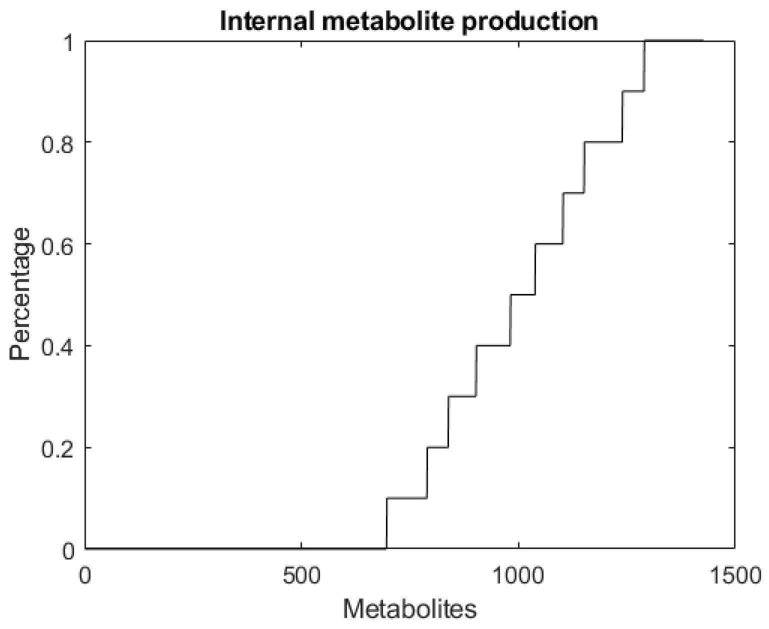

3.2. Visualization of the features of large-scale reconstruction resources and their subsets

We can also use the properties module in DEMETER to analyze and visualize the features of large-scale reconstruction resources, e.g., AGORA [1]. We can also do this for a subset of AGORA reconstructions. For instance, we can cluster all representatives of a given taxon, e.g., Bacteroidetes, by similarity. Note that this part of the tutorial is time-consuming.

Download all AGORA reconstructions.

```
system('curl -LJO
https://github.com/VirtualMetabolicHuman/AGORA/archive/master.zip')
unzip('AGORA-master')
modPath = [pwd filesep 'AGORA-master' filesep 'CurrentVersion' filesep
'AGORA_1_03' filesep' 'AGORA_1_03_mat'];
```

Define the path to the file with taxonomic information on AGORA.

```
infoFilePath = 'AGORA_infoFile.xlsx';
```

Define the column header which contain the information that should be used to extract a subset.

```
subHeader = 'Phylum';
```

Define the feature for which the subset of reconstructions should be extracted.

```
subFeature = 'Bacteroidetes';
```

Define the folder where the results from the extracted subset should be saved (optional, default folder will be used otherwise).

```
subsetFolder = [pwd filesep 'extractedModels'];
```

Run the extraction of the subset.

```
[extractedSubset,subsetFolder] = extractReconstructionResourceSubset(modPath,
infoFilePath, subHeader, subFeature, subsetFolder);
```

Define the folder where the results from the extracted subset will be saved (optional).

```
subsetPropertiesFolder = [pwd filesep 'subsetProperties'];
```

Define a name for the subset (optional).

```
subsetVersion = 'Bacteroidetes';
```

We will now compute the properties of the extracted reconstruction subset. If the subset contains enough reconstructions, tSNE plots, a visual representation of the similarity between the reconstructed strains, are also generated.

```
propertiesFolder = computeModelProperties(subsetFolder, infoFilePath,
subsetVersion, 'numWorkers', numWorkers, 'propertiesFolder',
subsetPropertiesFolder);
```

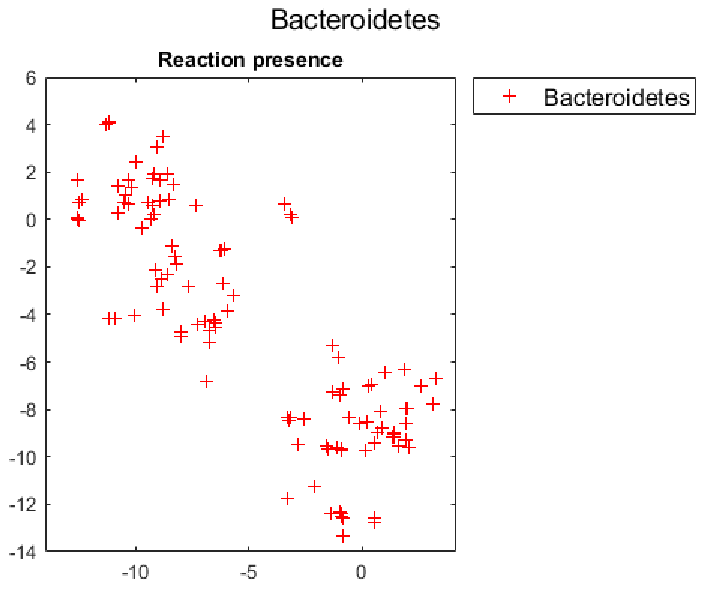

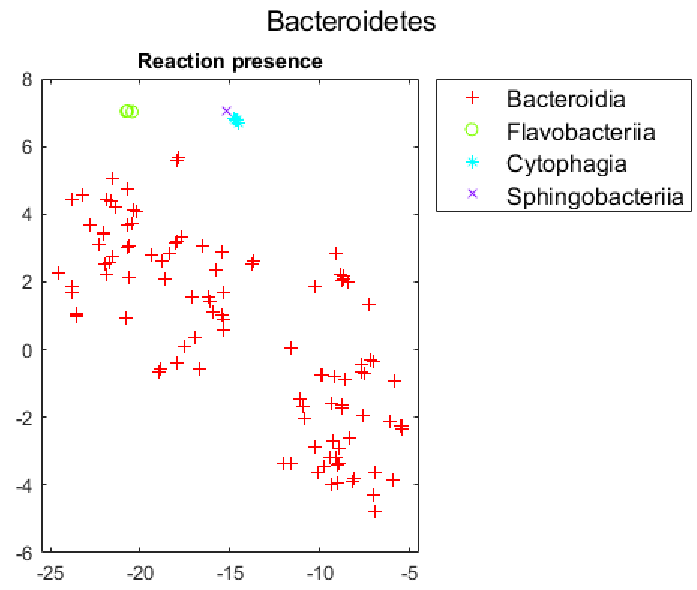

Bacteroidetes
Reaction presence
Bacteroidia
Flavobacteriia
Cytophagia
Sphingobacteriia

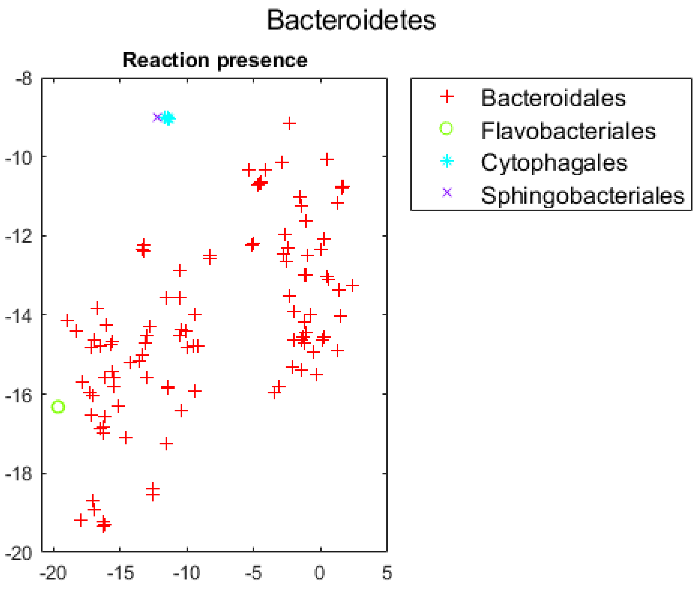

Bacteroidetes
Reaction presence
Bacteroidales
Flavobacteriales
Cytophagales
Sphingobacteriales

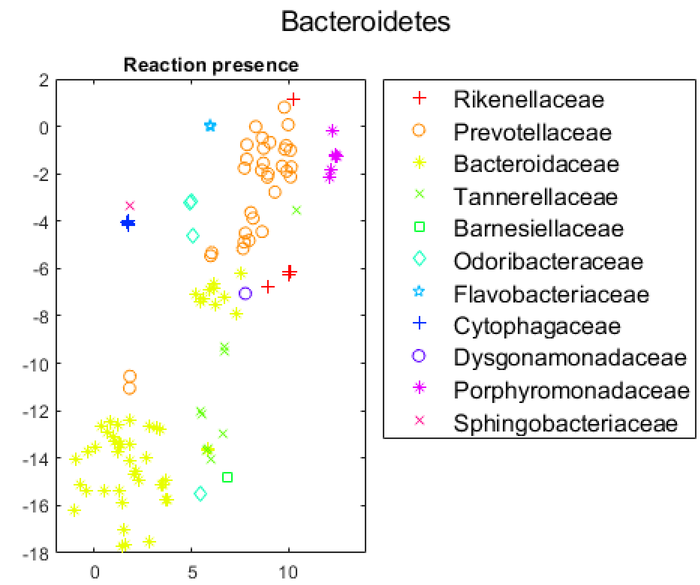
Bacteroidetes
Reaction presence
Rikenellaceae
Prevotellaceae
Bacteroidaceae
Tannerellaceae
Barnesiellaceae
Odoribacteraceae
Flavobacteriaceae
Cytophagaceae
Dysgonamonadaceae
Porphyromonadaceae
Sphingobacteriaceae

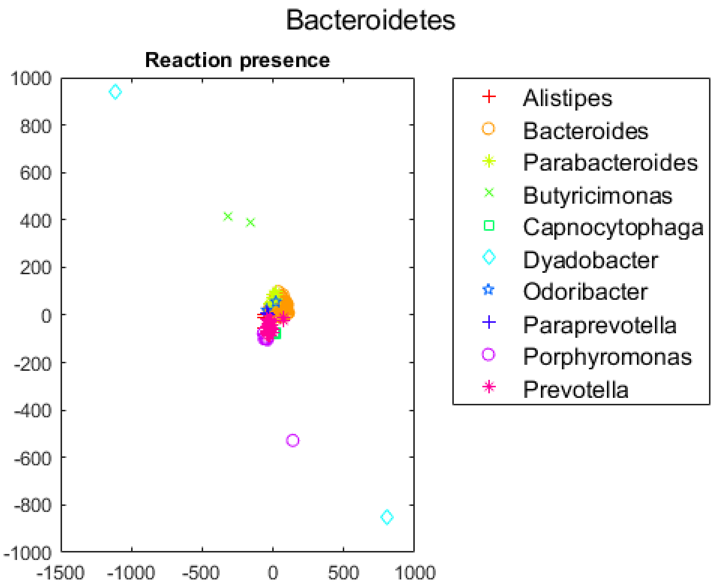
Bacteroidetes
Reaction presence
Alistipes
Bacteroides
Parabacteroides
Butyricimonas
Capnocytophaga
Dyadobacter
Odoribacter
Paraprevotella
Porphyromonas
Prevotella

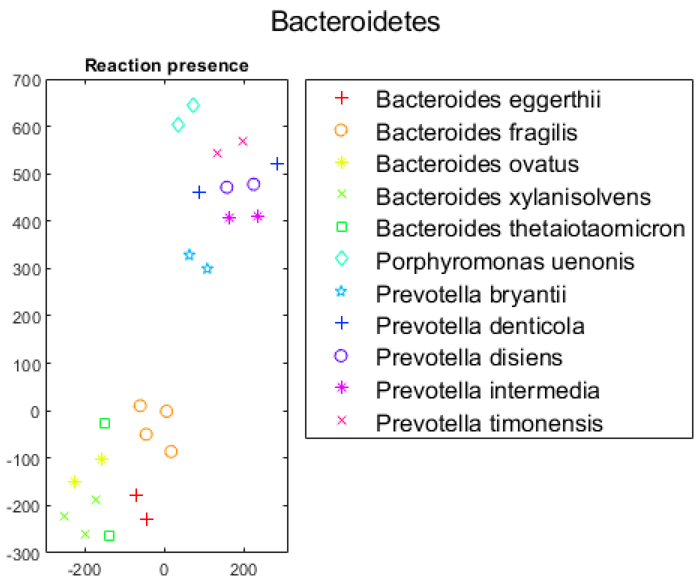
Bacteroidetes
Reaction presence
700
600
500
400
300
200
100
0
-100
-200
-300
-200
0
200
Bacteroides eggerthii
Bacteroides fragilis
Bacteroides ovatus
Bacteroides xylanisolvens
Bacteroides thetaiotaomicron
Porphyromonas uenonis
Prevotella bryantii
Prevotella denticola
Prevotella disiens
Prevotella intermedia
Prevotella timonensis

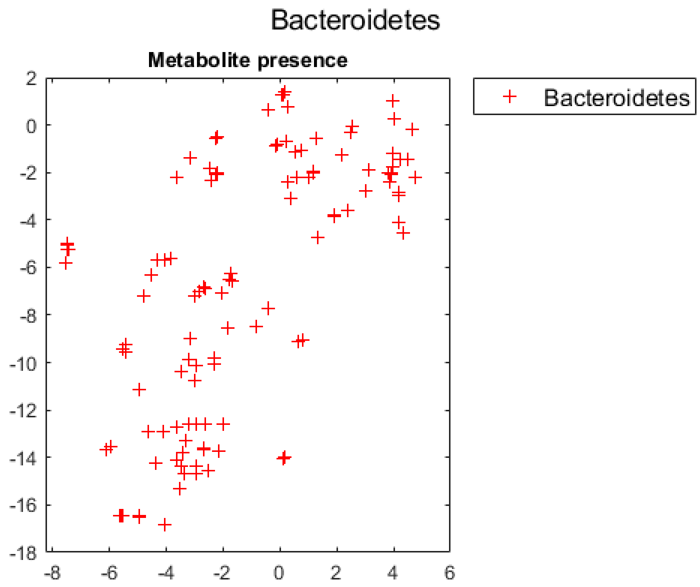
Bacteroidetes
Metabolite presence
2
0
-2
-4
-6
-8
-10
-12
-14
-16
-18
-8
-6
-4
-2
0
2
4
6
Bacteroidetes

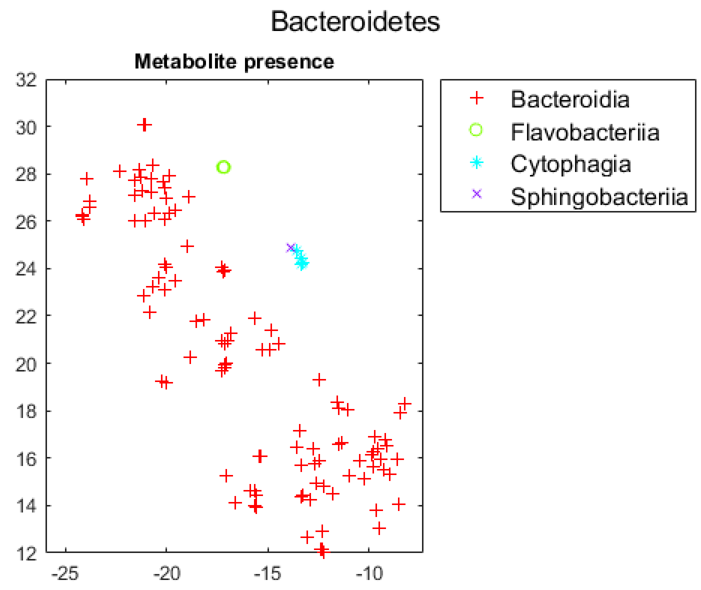

Bacteroidetes
Metabolite presence
32
30
28
26
24
22
20
18
16
14
12
-25
-20
-15
-10
Bacteroidia
Flavobacteriia
Cytophagia
Sphingobacteriia

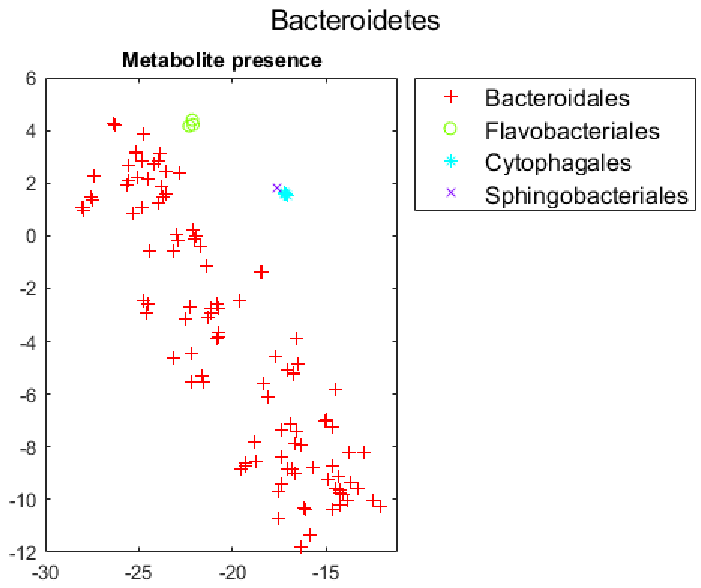

Bacteroidetes
Metabolite presence
6
4
2
0
-2
-4
-6
-8
-10
-12
-30
-25
-20
-15
Bacteroidales
Flavobacteriales
Cytophagales
Sphingobacteriales

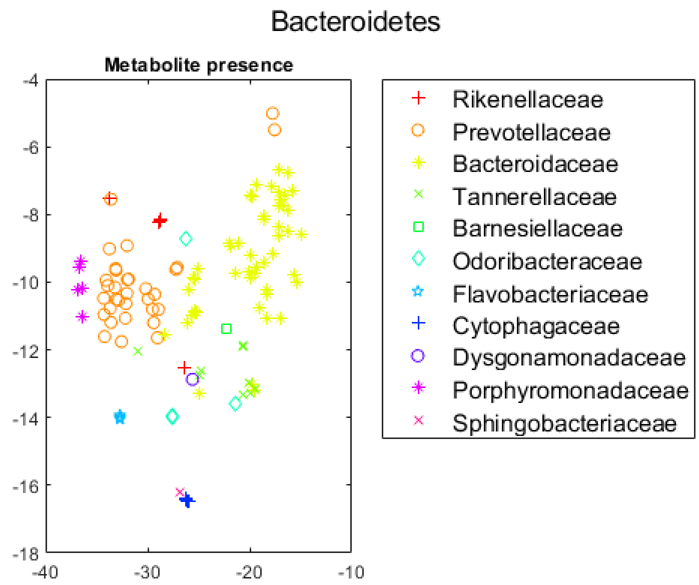
Bacteroidetes
Metabolite presence
-4
-6
-8
-10
-12
-14
-16
-18
-40
-30
-20
-10
Rikenellaceae
Prevotellaceae
Bacteroidaceae
Tannerellaceae
Barnesiellaceae
Odoribacteraceae
Flavobacteriaceae
Cytophagaceae
Dysgonamonadaceae
Porphyromonadaceae
Sphingobacteriaceae

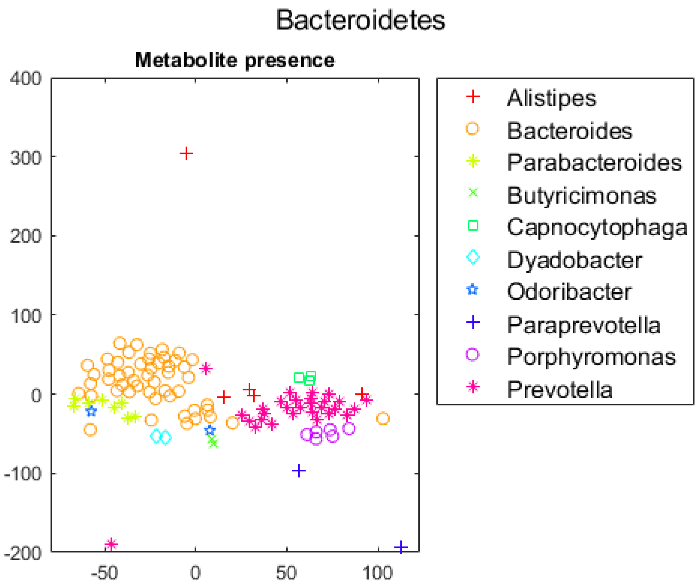
Bacteroidetes
Metabolite presence
400
300
200
100
0
-100
-200
-50
0
50
100
Alistipes
Bacteroides
Parabacteroides
Butyricimonas
Capnocytophaga
Dyadobacter
Odoribacter
Paraprevotella
Porphyromonas
Prevotella

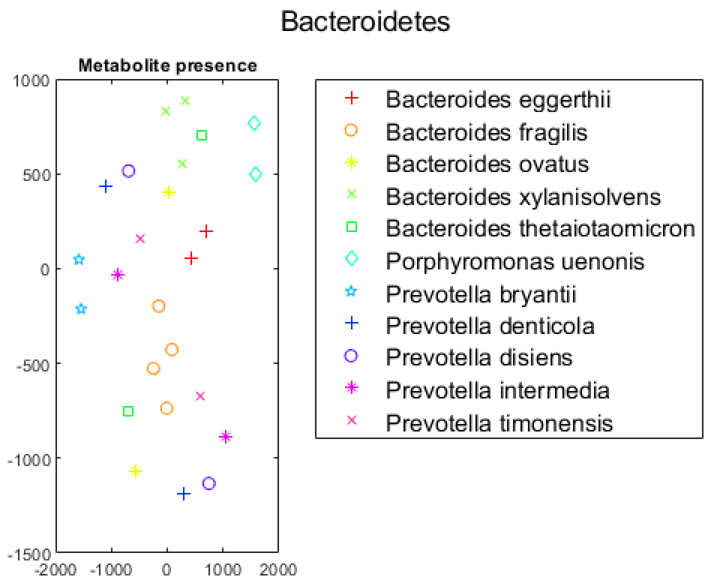
Bacteroidetes
Metabolite presence
1000
500
0
-500
-1000
-1500
-2000
-1000
0
1000
2000
Bacteroides eggerthii
Bacteroides fragilis
Bacteroides ovatus
Bacteroides xylanisolvens
Bacteroides thetaiotaomicron
Porphyromonas uenonis
Prevotella bryantii
Prevotella denticola
Prevotella disiens
Prevotella intermedia
Prevotella timonensis

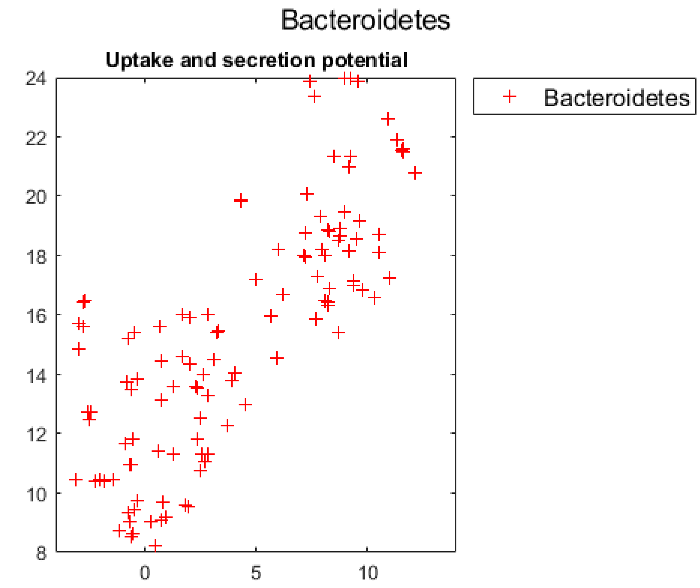
Bacteroidetes
Uptake and secretion potential
24
22
20
18
16
14
12
10
8
0
5
10
Bacteroidetes

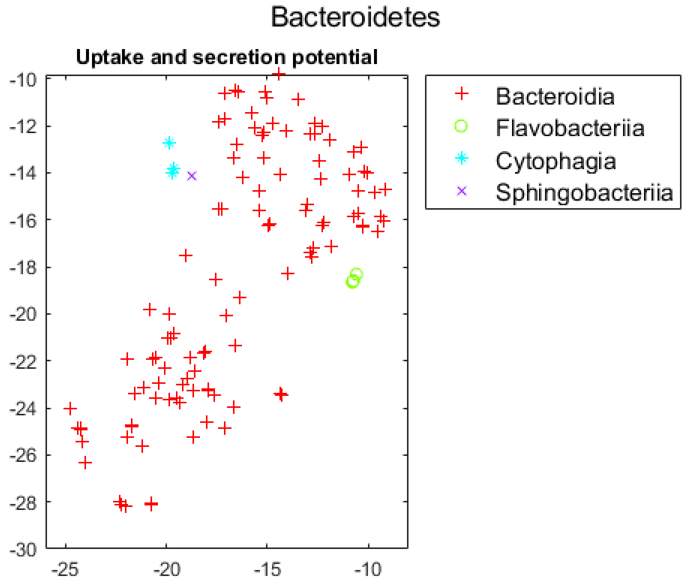
Bacteroidetes
Uptake and secretion potential
-10
-12
-14
-16
-18
-20
-22
-24
-26
-28
-30
-25
-20
-15
-10
Bacteroidia
Flavobacteriia
Cytophagia
Sphingobacteriia

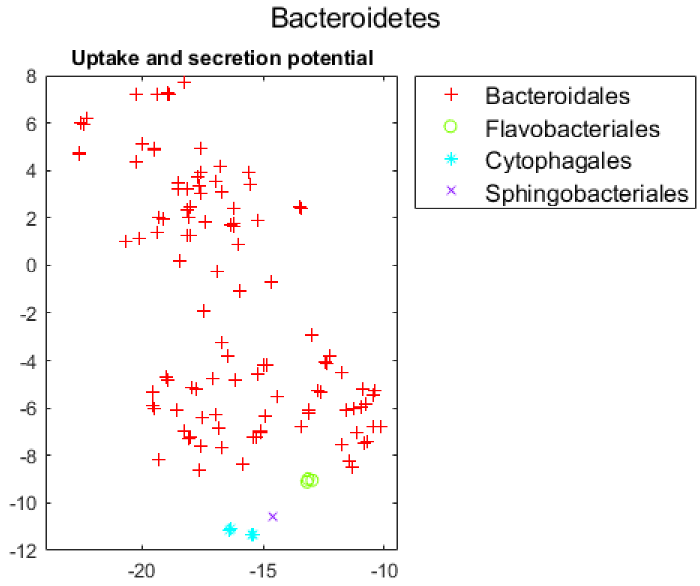
Bacteroidetes
Uptake and secretion potential
8
6
4
2
0
-2
-4
-6
-8
-10
-12
-20
-15
-10
Bacteroidales
Flavobacteriales
Cytophagales
Sphingobacteriales

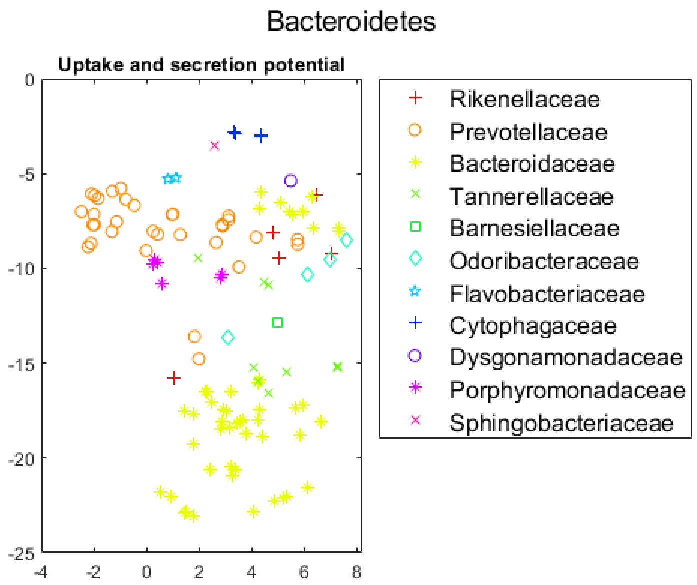
Bacteroidetes
Uptake and secretion potential
0
-5
-10
-15
-20
-25
-4
-2
0
2
4
6
8
Rikenellaceae
Prevotellaceae
Bacteroidaceae
Tannerellaceae
Barnesiellaceae
Odoribacteraceae
Flavobacteriaceae
Cytophagaceae
Dysgonamonadaceae
Porphyromonadaceae
Sphingobacteriaceae

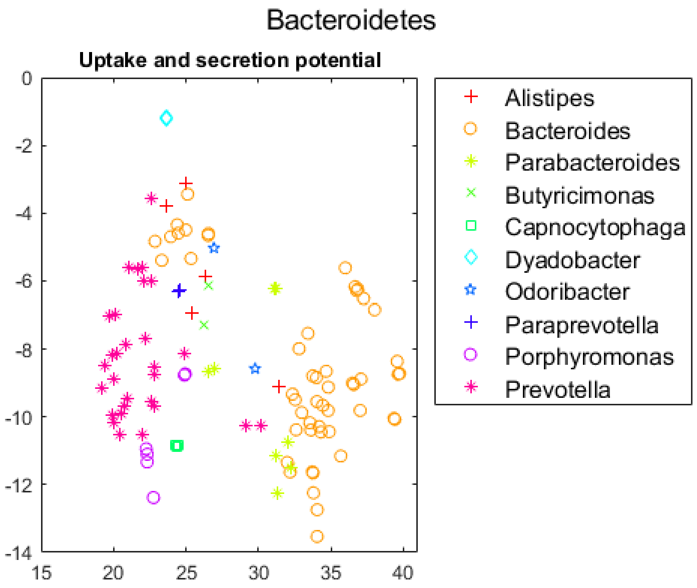
Bacteroidetes
Uptake and secretion potential
0
-2
-4
-6
-8
-10
-12
-14
15
20
25
30
35
40
Alistipes
Bacteroides
Parabacteroides
Butyricimonas
Capnocytophaga
Dyadobacter
Odoribacter
Paraprevotella
Porphyromonas
Prevotella

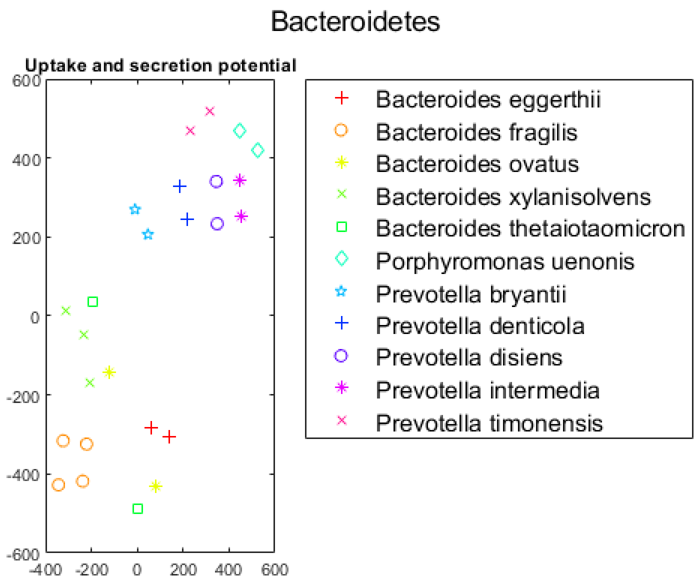
Bacteroidetes
Uptake and secretion potential
600
400
200
0
-200
-400
-600
-400
-200
0
200
400
600
Bacteroides eggerthii
Bacteroides fragilis
Bacteroides ovatus
Bacteroides xylanisolvens
Bacteroides thetaiotaomicron
Porphyromonas uenonis
Prevotella bryantii
Prevotella denticola
Prevotella disiens
Prevotella intermedia
Prevotella timonensis

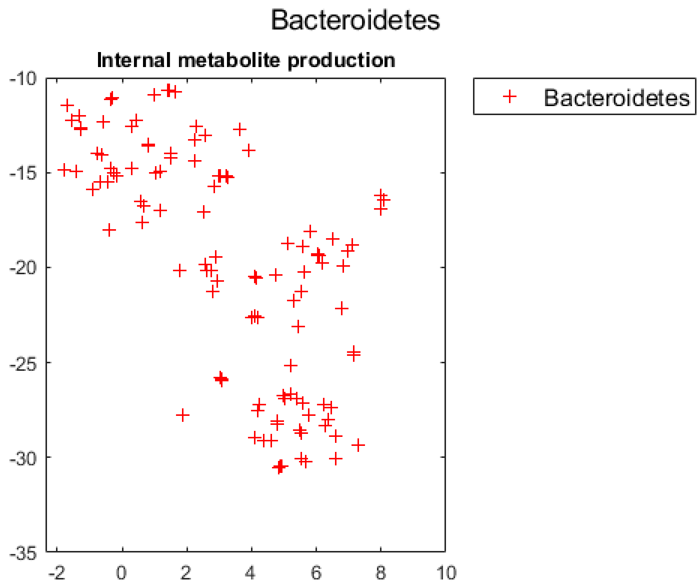
Bacteroidetes
Internal metabolite production
-10
-15
-20
-25
-30
-35
-2
0
2
4
6
8
10
Bacteroidetes

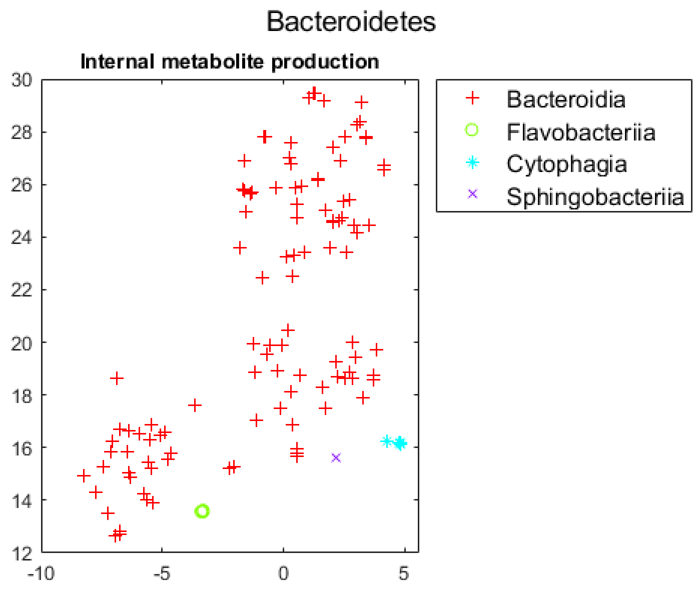

Bacteroidetes
Internal metabolite production
Bacteroidia
Flavobacteriia
Cytophagia
Sphingobacteriia
30
28
26
24
22
20
18
16
14
12
-10
-5
0
5

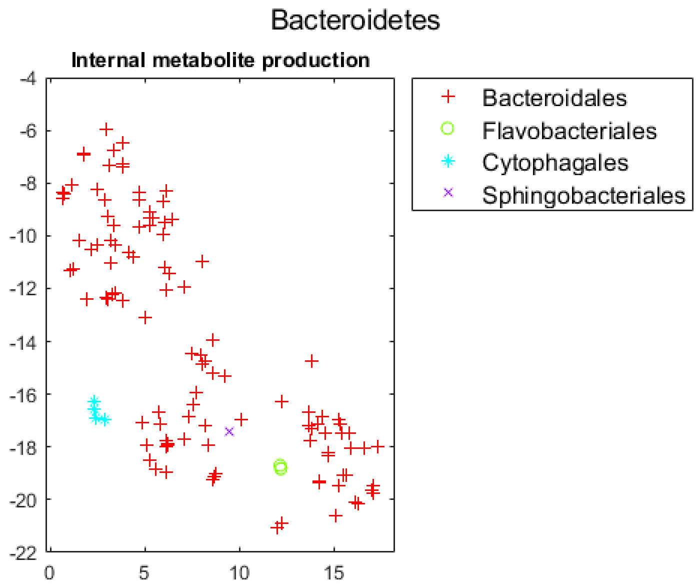

Bacteroidetes
Internal metabolite production
Bacteroidales
Flavobacteriales
Cytophagales
Sphingobacteriales
-4
-6
-8
-10
-12
-14
-16
-18
-20
-22
0
5
10
15

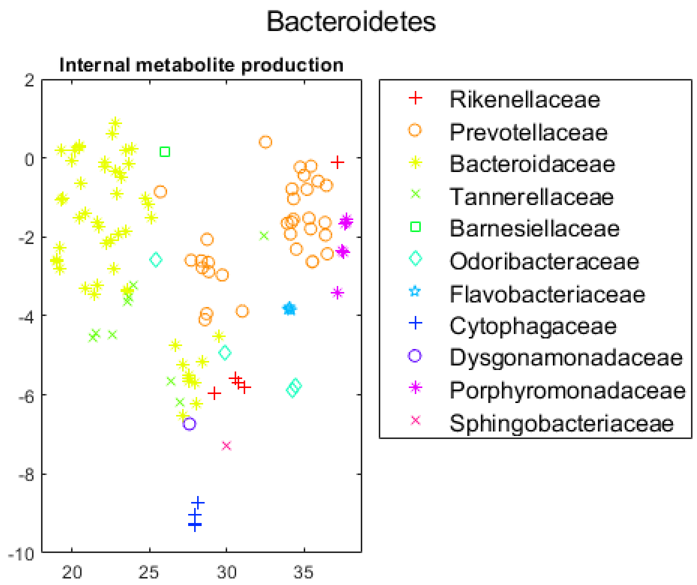
Bacteroidetes
Internal metabolite production
2
0
-2
-4
-6
-8
-10
20
25
30
35
Rikenellaceae
Prevotellaceae
Bacteroidaceae
Tannerellaceae
Barnesiellaceae
Odoribacteraceae
Flavobacteriaceae
Cytophagaceae
Dysgonamonadaceae
Porphyromonadaceae
Sphingobacteriaceae

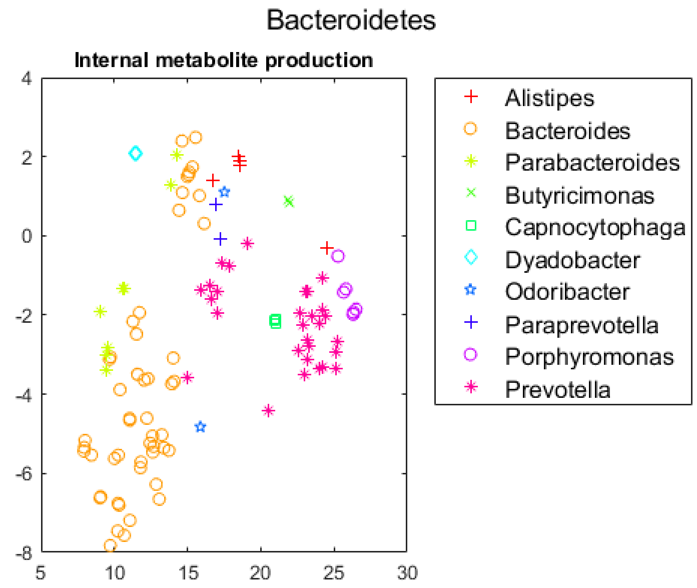
Bacteroidetes
Internal metabolite production
4
2
0
-2
-4
-6
-8
5
10
15
20
25
30
Alistipes
Bacteroides
Parabacteroides
Butyricimonas
Capnocytophaga
Dyadobacter
Odoribacter
Paraprevotella
Porphyromonas
Prevotella

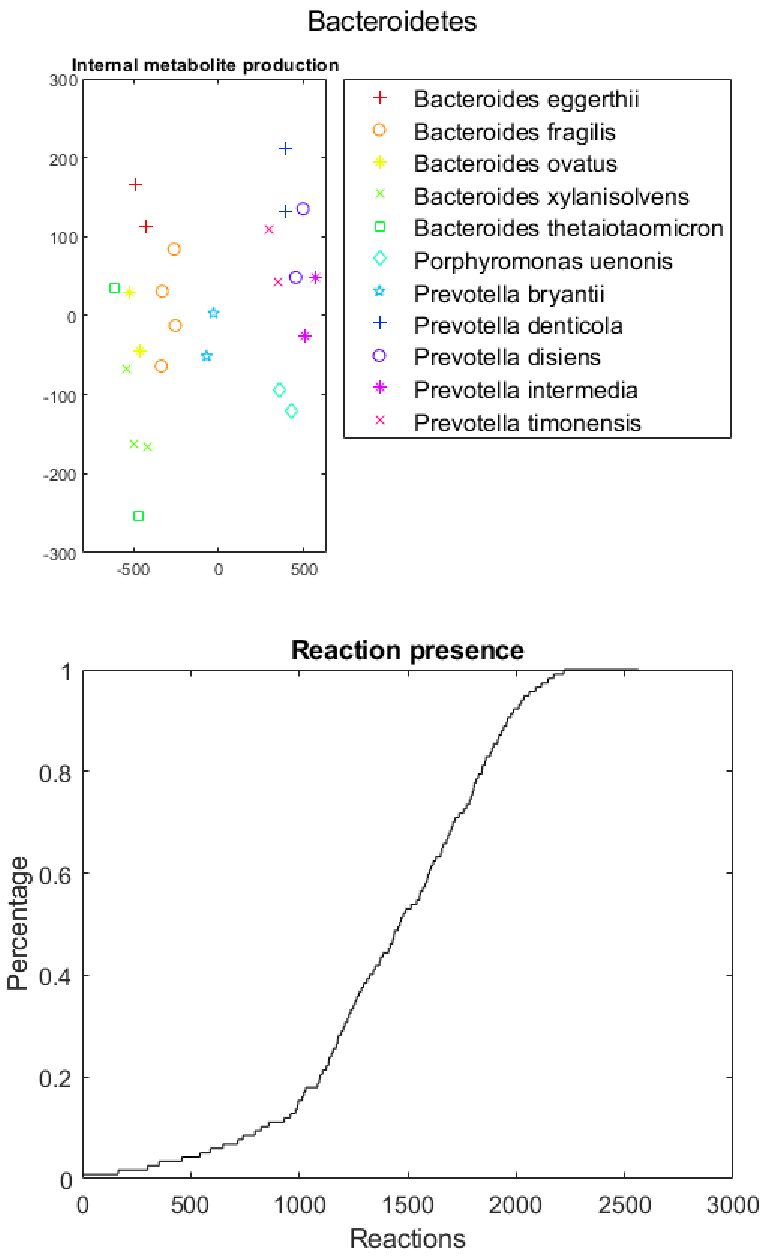
Bacteroidetes
Internal metabolite production
Bacteroides eggerthii
Bacteroides fragilis
Bacteroides ovatus
Bacteroides xylanisolvens
Bacteroides thetaiotaomicron
Porphyromonas uenonis
Prevotella bryantii
Prevotella denticola
Prevotella disiens
Prevotella intermedia
Prevotella timonensis
Reaction presence
Percentage
Reactions

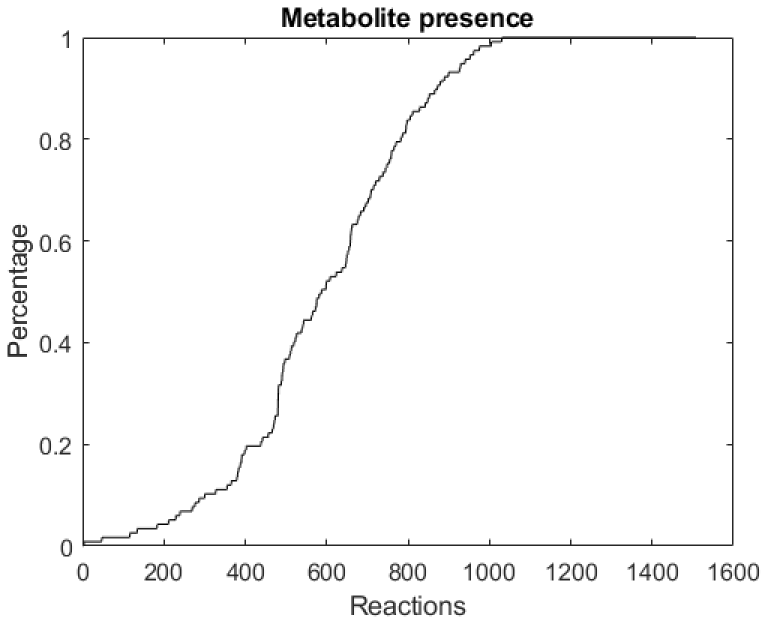

Metabolite presence
Percentage
Reactions
0
0.2
0.4
0.6
0.8
1
0
200
400
600
800
1000
1200
1400
1600

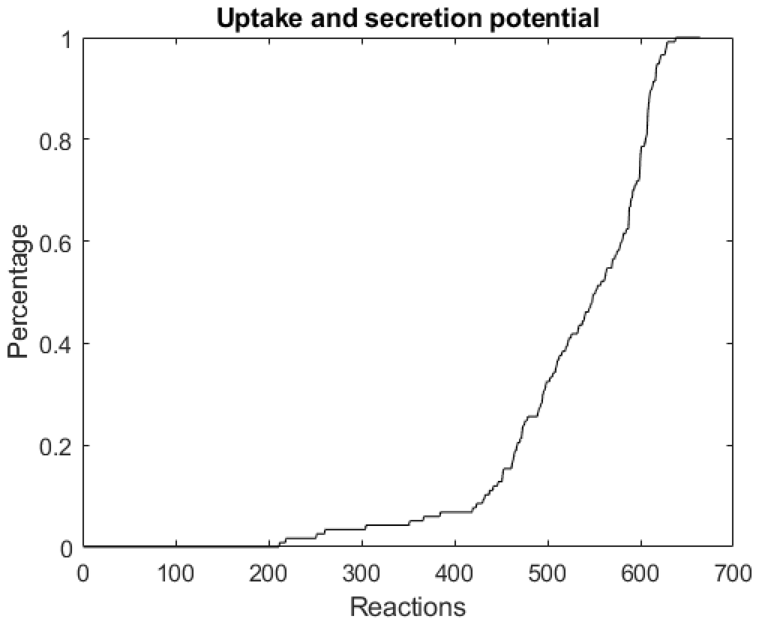

Uptake and secretion potential
Percentage
Reactions
0
0.2
0.4
0.6
0.8
1
0
100
200
300
400
500
600
700

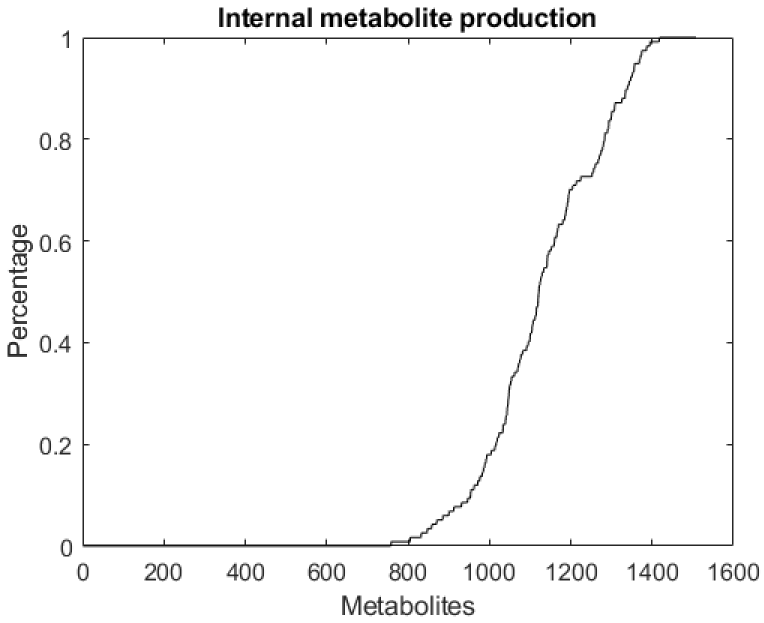

References

[1] Heinken A, Magnúsdóttir S, Fleming R.M.T., Thiele I, DEMETER: Efficient simultaneous curation of genome-scale reconstructions guided by experimental data and refined gene annotations. arXiv (2021)

[2] Magnúsdóttir S, Heinken A, Kutt L, Ravcheev DA et al, Generation of genome-scale metabolic reconstructions for 773 members of the human gut microbiota. Nat Biotechnol. 35(1):81-89 (2017).

[3] Heinken A, Acharya G, Ravcheev DA, Hertel J et al, AGORA2: Large scale reconstruction of the microbiome highlights wide-spread drug-metabolising capacities. bioRxiv (2020).

[4] Chen IA, Chu K, Palaniappan K, Ratner A et al, The IMG/M data management and analysis system v.6.0: new tools and advanced capabilities. Nucleic Acids Res. 49(D1):D751-D763 (2021).

[5] Noronha A, Modamio J, Jarosz Y, Guerard E et al., The Virtual Metabolic Human database: integrating human and gut microbiome metabolism with nutrition and disease. Nucleic Acids Res 47(D1):D614-D624 (2019).

[6] Thorleifsson SG, Thiele I, rBioNet: A COBRA toolbox extension for reconstructing high-quality biochemical networks. Bioinformatics 27(14):2009-10 (2010).

[7] Kanehisa M, Furumichi M, Sato Y, Ishiguro-Watanabe M, Tanabe M, KEGG: integrating viruses and cellular organisms. Nucleic Acids Res. 49(D1):D545-D551 (2021).

[8] Wishart DS, Feunang YD, Marcu A, Guo AC, et al., HMDB 4.0 — The Human Metabolome Database for 2018. Nucleic Acids Res. 46(D1):D608-17 (2018).

[9] Thiele I, Palsson BØ, A protocol for generating a high-quality genome-scale metabolic reconstruction. Nat Protoc 5(1):93-121 (2010).
